# Conformational Properties of Polymers at Droplet Interfaces as Model Systems for Disordered Proteins

**DOI:** 10.1021/acsmacrolett.3c00456

**Published:** 2023-10-19

**Authors:** Jiahui Wang, Dinesh Sundaravadivelu Devarajan, Arash Nikoubashman, Jeetain Mittal

**Affiliations:** † Artie McFerrin Department of Chemical Engineering, Texas A&M University, College Station, Texas 77843, United States; ‡ Institute of Physics, Johannes Gutenberg University Mainz, Staudingerweg 7, 55128 Mainz, Germany; § Leibniz-Institut für Polymerforschung Dresden e.V., Hohe Straße 6, 01069 Dresden, Germany; ∥ Institut für Theoretische Physik, Technische Universität Dresden, 01069 Dresden, Germany; ⊥ Department of Chemistry, Texas A&M University, College Station, Texas 77843, United States; # Interdisciplinary Graduate Program in Genetics and Genomics, Texas A&M University, College Station, Texas 77843, United States

## Abstract

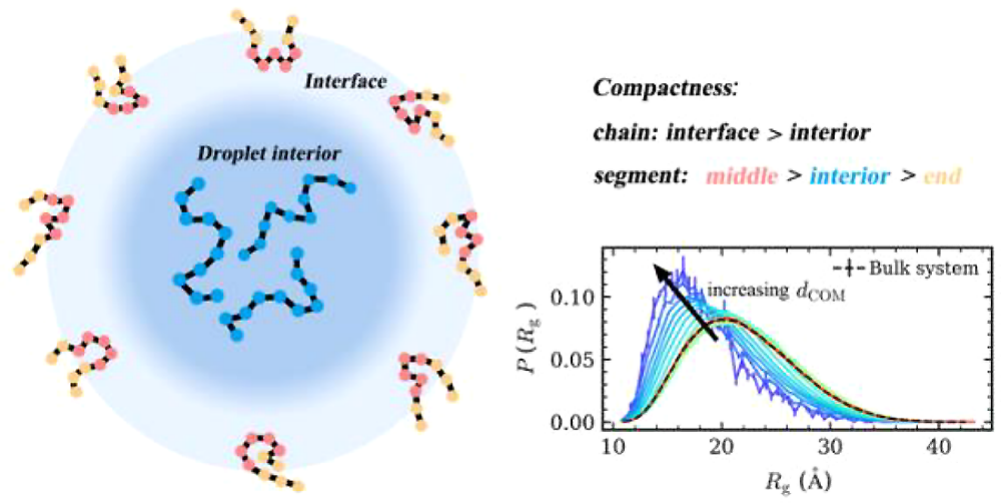

Polymer models serve as useful tools for studying the formation and physical properties of biomolecular condensates. In recent years, the interface dividing the dense and dilute phases of condensates has been discovered to be closely related to their functionality, but the conformational preferences of the constituent proteins remain unclear. To elucidate this, we perform molecular simulations of a droplet formed by phase separation of homopolymers as a surrogate model for the prion-like low-complexity domains. By systematically analyzing the polymer conformations at different locations in the droplet, we find that the chains become compact at the droplet interface compared with the droplet interior. Further, segmental analysis revealed that the end sections of the chains are enriched at the interface to maximize conformational entropy and are more expanded than the middle sections of the chains. We find that the majority of chain segments lie tangential to the droplet surface, and only the chain ends tend to align perpendicular to the interface. These trends also hold for the natural proteins FUS LC and LAF-1 RGG, which exhibit more compact chain conformations at the interface compared to the droplet interior. Our findings provide important insights into the interfacial properties of biomolecular condensates and highlight the value of using simple polymer physics models to understand the underlying mechanisms.

Membraneless organelles or biomolecular condensates formed through liquid*–*liquid phase separation (LLPS) have been widely reported in various cellular functions, including gene expression, signal transduction, stress response, and the assembly of macromolecular complexes.^1−5^ Examples of such condensates include the nucleolus, Cajal bodies, P bodies, and stress granules. Intrinsically disordered proteins (IDPs) play an important role in the formation of biomolecular condensates through LLPS.^6−11^ Due to the numerous similarities between IDPs and synthetic polymers, classical polymer models offer a powerful approach for investigating the conformations,^12^ dynamics,^13^ and phase behavior of IDPs.^14−17^ In particular, such models have been extensively used to reveal the sequence-dependent conformations of IDPs in solution and in condensates.^18−27^

In recent years, there has been increasing recognition of the important role played by the interfaces that divide the dense and dilute phases of biomolecular condensates. Folkmann et al. have reported that the IDP assemblies at the interface of PGL-3 droplets reduced the surface tension, thus preventing droplet coarsening. Kelley et al. reported that amphiphilic proteins on the surface of condensates acted like surfactants, thus regulating the size and structure of condensates.^28^ Further, the interface can affect the interactions between biomolecular condensates and other biomolecules within the cell.^29^ Böddeker et al. demonstrated that cytoskeletal filaments had a nonspecific affinity for stress granule interfaces, which explained the distinct enhancement of tubulin density around granules.^30^ Lipiński et al. reported that the condensate interface can serve as a nucleation site promoting the aggregation of amyloidogenic proteins.^31^ Inspired by the critical role of interfaces in various functionalities, much research has focused on determining mesoscopic interfacial properties such as surface tension,^13,32,33^ surface adsorption,^34^ and electrochemical properties,^35^ which are deemed important for regulating biological functions.^29,36^ For example, it was shown that the condensates could form an autophagosome only if the surface tension is lower than a critical value.^37^ The film formed by protein adsorption at the air/water interface has a low interfacial elasticity at the protein’s isoelectric pH because of the compact structure.^38^

However, the microscopic conformations of individual IDPs at interfaces remain largely unclear, even though these exposed polymers dictate the surface properties. For example, surfaces grafted by a synthetic polymer such as PNIPAM have been shown to result in a 4-fold difference in the Young’s modulus between the swollen and collapsed states,^39^ and the surface wettability is stronger for polymer interfaces in stretched states than in collapsed states.^40^ Conformations adopted within condensates can have implications for controlling the functional state of biomolecules.^41^ In that regard, Farag et al. studied the interfacial conformations of biomolecular condensates using lattice-based Monte Carlo simulations.^42,43^ They reported that the overall dimensions of prion-like low-complexity domains and homopolymers varied within the condensates, being most *expanded* at the interface and preferring to be oriented *perpendicular* to it. These pioneering studies open up avenues for detailed characterization of the segmental-level conformational properties and their contribution to the chain-level conformational preferences at the condensate interfaces, which remain elusive.

In this article, we report chain-level and segmental-level conformations of IDPs at the condensate interface from molecular simulations of hydrophobic homopolymers and two naturally occurring IDPs. We use an off-lattice polymer model, where each residue (“monomer”) is represented by a spherical bead in an implicit solvent (model details are provided as Supporting Information (SI)). This model is a good approximation for numerous IDPs, such as prion-like low-complexity domains.^42^ Recently, we successfully used hydrophobic homopolymers as a reference to establish the biophysics of phase separation of IDPs, revealing that distributed interactions better stabilize the condensed phase than localized interactions.^24^ To understand the basic principles governing the chain conformations in the droplet, we focus here on hydrophobic homopolymers.

Due to the hydrophobic nature of the employed model IDP, it quickly formed a spherical condensate of radius *R* = 249 Å, which is defined as the position where the concentration drops to half the value near the droplet center (Figure 1). The droplet maintains a spherical shape (Figure S1), so that we neglect the influence of shape fluctuations on the spatial dependence of chain conformations in the droplet. Drawing upon previous research on the liquid–vapor interface in slabs,^44^ the radial concentration profile is fit to a hyperbolic tangent functional form
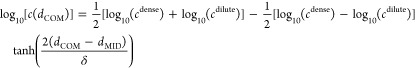
1where *d*_COM_ is the distance from the droplet’s center of mass, *c*(*d*_COM_) is the radial monomer concentration, *c*^dense^ and *c*^dilute^ are the monomer concentrations of the dense phase and dilute phase, respectively, *d*_MID_ is the midpoint of the interface, and δ is the width of the interface. By fitting the computed radial concentration curve to eq 1, the interfacial region was defined (Figure 1). To establish a bulk reference without interface effects, we simulated a concentrated homopolymer solution in a cubic box at a constant pressure of *P* = 0 atm. In the absence of external pressure (i.e., *P* = 0 atm), the system’s volume changed in response to the polymer–polymer interactions to reach a naturally preferred concentration, which would be the same as the dense phase concentration obtained from a phase coexistence simulation.^13^ With increasing *d*_COM_, the concentration in the droplet interior remained constant (equal to the expected concentration in the bulk system), while it monotonically decreased at the interface. In this case, the dilute phase concentration is equal to 0 mM, as all chains are inside the droplet.

**Figure 1:**
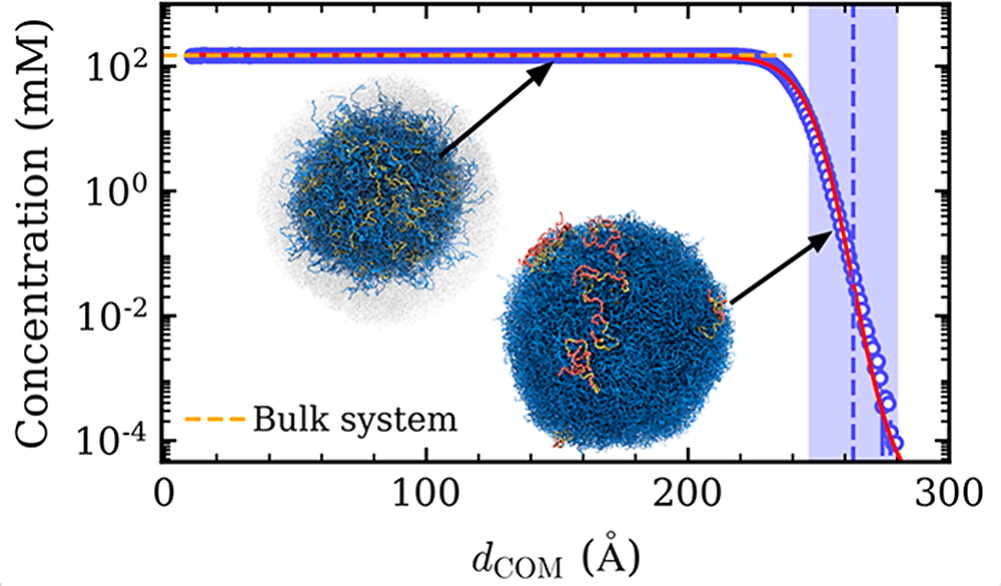
Concentration as a function of the distance from the droplet’s center of mass (*d*_COM_). The horizontal dashed line represents the concentration in the bulk system. The red line is the fitted curve for the simulation data (symbols). The purple shaded area is the interface region. The vertical dashed line represents the middle of the interface. The inset shows simulation snapshots of the droplet interior and interface. The yellow chains in the interior snapshot are at a distance of *d*_COM_ = 150 Å. The chains colored yellow and red in the interface snapshot have their center-of-mass in the interface, with the red beads representing segments in the interface.

After determining the droplet interface, we studied the conformations of polymer chains within the interface and the dense phase of the droplet. For this purpose, we computed the radius of gyration of the chains^45^
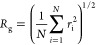
2where the sum goes over the *N* monomers in the chain, and *r*_i_ is the distance from the *i*^th^ monomer to the chain’s center of mass (COM). We find that the corresponding *R*_g_ distribution in the bulk system (Figure 2) was significantly broader, as compared to that of the single chain (Figure S2), highlighting the preference of chains to expand inside the condensate, as expected from Flory’s ideality hypothesis.^46^ Next, we analyzed the distribution of *R*_g_ at different positions within the droplet. Based on the distance between the chain’s COM and the droplet’s COM (*d*_COM_), we assigned the polymer chains into different positional bins (Figure 2a). We found that when *d*_COM_ ≤ 210 Å, where the local monomer concentration is equal to the bulk system concentration (Figure 1), the *R*_g_ distributions overlapped with the bulk distribution, indicating the consistency between the interior of the droplet and the bulk phase. With increasing *d*_COM_, the peak of the *R*_g_ distribution shifted gradually to smaller *R*_g_ values (Figure 2a), demonstrating a continuous transition from expanded conformations to more compact conformations when moving from the interior of the droplet toward the interface. Further, we characterized the average number of interchain and intrachain contacts for each monomer within the droplet (Figure 2b), which we found to decrease monotonically with increasing *d*_COM_ in the interface region because of decreasing monomer concentration (Figure 1). Though the overall number of contacts at the interface decreased, the number of average intrachain contacts was always larger than the number of interchain contacts (Figure 2b), which is consistent with the preference for compact conformations at the interface rather than in the droplet interior (Figure 2a), where interchain interactions dominated.

**Figure 2:**
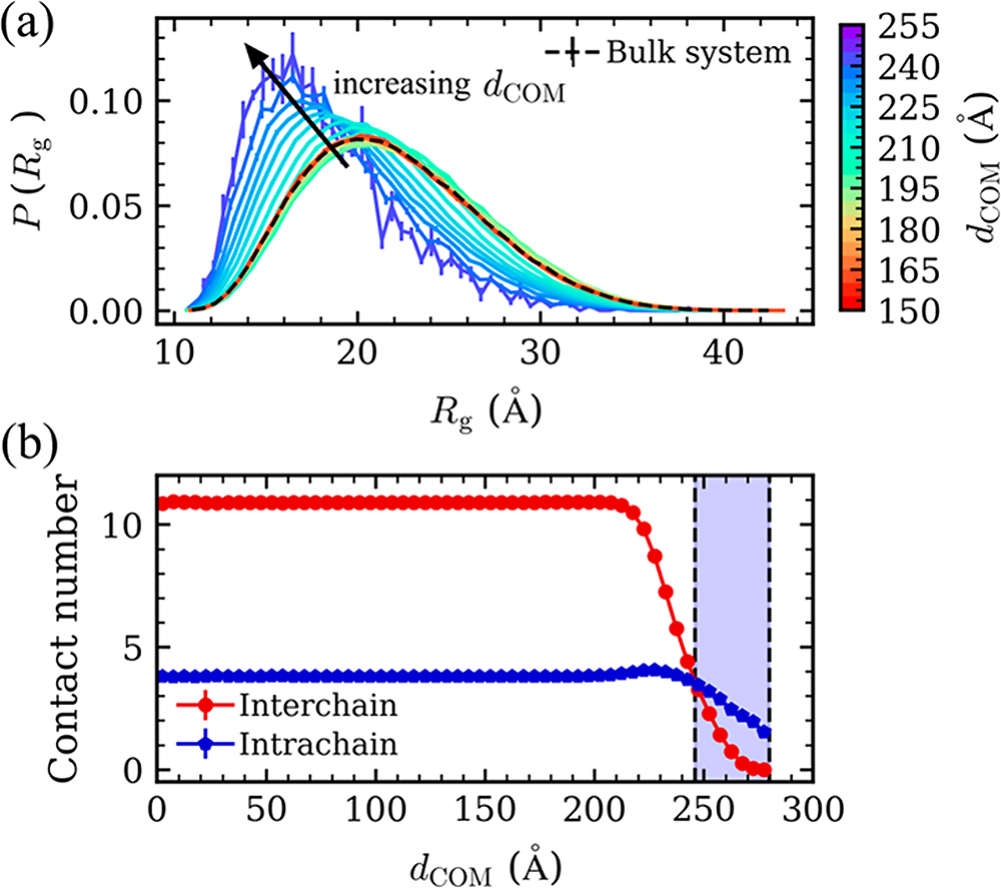
(a) Distribution of the radius of gyration (*R*_g_). The dashed line represents the *R*_g_ distribution in a bulk system. The color gradient from red to purple corresponds to the distribution of *R*_g_ for chains with *d*_COM_ ranging from 150 to 255 Å. (b) Average contact number per monomer as a function of *d*_COM_ for the inter- and intrachain contacts. The vertical dashed lines represent the interface boundaries.

Our observation of compact chain-level conformations at the interface led us to investigate how segments within a chain contributed to its compaction, following previous calculations for the end-to-end vector of grafted polymers^47^ and *R*_g_ of polymer thin films.^48^ We first characterized the distribution of chain segments of different lengths *n*_seg_ at the droplet interface (Figure 3a,b). The middle bead of the segments for a given *n*_seg_ serves as the reference bead, the index of which we refer to as the segment index. Note that a segment index closer to 1 or 50 corresponds to a segment closer to the chain termini, and the curves are symmetrical because the homopolymer chains do not have any “heads” or “tails”. We found that the highest probability always occurred at the position closest to the chain’s ends at the interface, regardless of the length of the segment, suggesting that the chain ends were more inclined to distribute at the interface compared to the middle of the chains. This is in stark contrast to the droplet interior, where we found the same probabilities for segments at different relative positions within the chain (Figure 3b). The enrichment of chain ends at the interface can be understood by considering the loss in conformational entropy incurred by placing a monomer at the interface, which is smaller for the end monomers compared with any other monomer.

**Figure 3:**
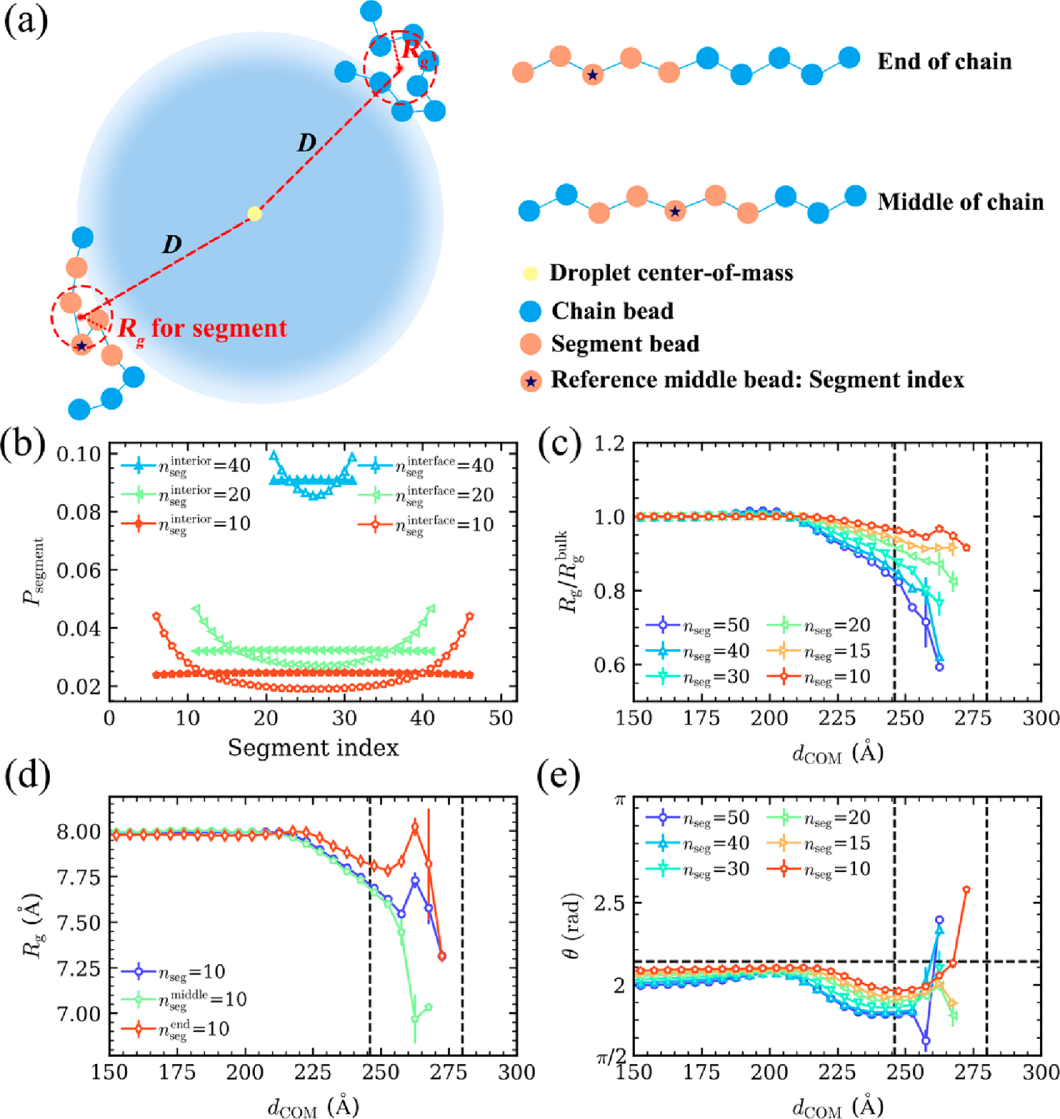
Segmental analysis. (a) Schematic diagram of the *R*_g_ calculation method for chains and their segments. Blue beads represent the chain monomers, while orange beads represent a specific segment. (b) Probability distribution of the segments for different segment lengths (*n*_seg_) at the interface (hollow symbols) and in the droplet interior (solid symbols). The segment index is represented by the index of the middle bead of each segment (reference bead). When *n*_seg_ is even, the larger index between the two middle beads is considered as the segment index. (c) Normalized average *R*_g_ of the segments of different length as a function of *d*_COM_. The black dashed lines represent the boundaries of the interface. (d) *R*_g_ of segments consisting of 10 monomers as a function of *d*_COM_. The purple line represents the average *R*_g_ for all segments in the chain of that length. The green and red lines represent the segments located at the middle and end of the chains, respectively. (e) Angle (θ) of the segments of different length as a function of *d*_COM_. The horizontal dashed line represents the average angle for the isotropic distribution of segments.

We next asked if the spatial preference of the chain segments at the droplet interface vs its interior had an effect on the segmental-level conformations. For this purpose, we calculated the average *R*_g_ for the segments of different lengths *n*_seg_ as a function of distance *d*_COM_ between their COM and the droplet’s COM (Figure 3c). We found that the *R*_g_ at the interface is smaller than that in the droplet interior for all of the *n*_seg_ considered. For *n*_seg_ > 15, *R*_g_ monotonically decreased as the droplet interface is approached. For *n*_seg_= 15, *R*_g_ no longer decreased in the interface region. Interestingly, for *n*_seg_ = 10, we found that *R*_g_ marginally increased in the middle of the interface region, following which it decreased again. To explain this nonmonotonic trend in *R*_g_ for the short segments at the interface, we analyzed *R*_g_ of different *n*_seg_ located only at the chain ends and compared it with that in the middle of the chain. For longer segments *n*_seg_ ≥ 20, the segmental *R*_g_ monotonically decreased from the droplet interior to the interface, regardless of the position of the segments (Figure S3). However, for shorter segments *n*_seg_ ≤ 15 (Figures 3d and S3), we found that the *R*_g_ of the segments located at the chain ends exhibited a nonmonotonic trend in the interface region: we observed an increase in segmental *R*_g_ for *d*_COM_ > 250 Å, attaining the maximum value in the middle of the interface region, following which it continued to decrease. Surprisingly, the *R*_g_ of shorter segments located in the middle of the chains monotonically decreased throughout the interface region. These observations indicate that the chain termini are the only contributors to the observed expansion of the short segments (Figure 3c). In summary, although the *whole* chain is more compact at the interface, short terminal segments are more expanded than the middle segments at the interface and even slightly more expanded than the short segments in the droplet interior.

Given the stark differences in the global and local conformations of the chains in the interface region, we next investigated their chain- and segmental-level orientations. We defined the angle θ between the segment-to-droplet COM vector and the segment end-to-end vector to characterize the orientation of the chains in the interface region (Figure S4a). The positions of the segments are again defined through *d*_COM_. When the angle is close to π/2, the segment lies tangential to the droplet surface, whereas an angle close to π corresponds to an orientation perpendicular to the droplet surface. For different segment lengths (*n*_seg_ = 10, 15, 20, 30, 40) and the whole chain (*n*_seg_ = 50), the angles show an increasing trend at the interface (Figure 3e). This increase primarily originates from the segments near the chain ends (Figure S4c) rather than segments in the middle of the chains (Figure S4b). This observation can be explained by considering the end-to-end distance (*R*_e_) (Figure S5). The smallest angle for segments (Figure S4a) can be estimated as
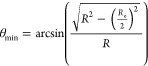
3where *R* is the distance between the terminal bead of the segment and the droplet COM, and *R*_e_ is the end-to-end distance of the segment. At the interface, *R* is much larger than *R*_e_, so that θ_min_ becomes close to π/2 (i.e., tangential orientation). If segments are oriented randomly, the average angle should be close to π–1 (calculation shown in SI). We found that the angles of segments located in the middle of chains were between π/2 and π–1, indicating that these segments lied predominantly tangential to the surface (Figure S4b). In contrast, end segments near the interface exhibited a tendency to form angles larger than π–1 (Figure S4c). However, these segments remained closer to the angle representing isotropic segments, π–1, than perpendicular segments, π. These results demonstrate that despite the tendency of chain ends to align perpendicular to the surface the majority of chain segments remain primarily tangential to the droplet surface.

Based on our investigation of the size and orientation of chain segments at different positions in the droplet, we propose a model for homopolymer conformations in condensates, as depicted in Figure 4. Compared with the droplet interior, which we found to be consistent with the bulk phase, the distribution of *R*_g_ in the interface shifted to smaller values, indicating more compact chain conformations at the droplet interface. Through our characterization of the conformational preferences of segments of different lengths at different positions, we have discovered that segments located at the chains’ ends expand slightly more in the droplet interface compared to the droplet interior, while the middle segments of the chains are more collapsed in the interface region than in the droplet interior. These end segments are enriched at the interface to minimize entropy loss during LLPS. At the interface, the chain ends exhibit a tendency to align perpendicularly to the interface but the majority of the chains are still parallel to the surface. We speculate that these conformational changes originate from the change in the local environment surrounding the chains: Compared to the interior of the droplet, the lower monomer concentration at the interface leads to a decrease in the (effective) solvent quality, thus, resulting in chain collapse. Similar compact chain conformations at the interface have been reported previously for hydrophobic homopolymers.^49−51^

**Figure 4:**
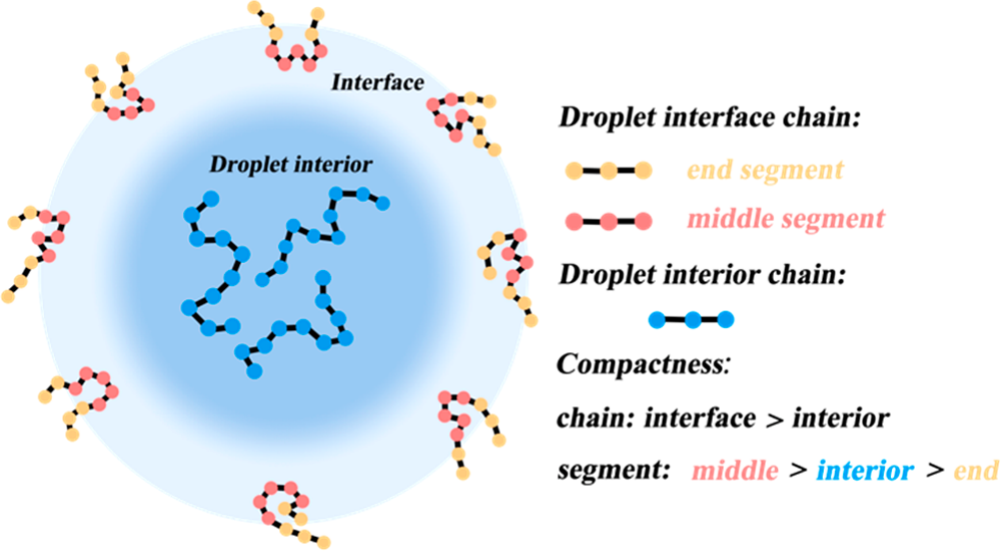
Schematic of homopolymer conformations at the droplet interface. Polymer chains are more compact at the interface than in the droplet interior. Compared to the interior of the droplet, the chain ends are more expanded, while the middle segments of the chains are more collapsed at the interface. The chain ends exhibit a tendency to be perpendicular to the droplet surface, whereas the majority of chains lie predominantly tangential to the droplet surface.

Given that our findings illustrate the conformational preferences of homopolymers at their droplet interface, we next wanted to investigate how our observations translate to the condensates formed by naturally occurring IDPs. To this end, we conducted simulations on FUS LC and LAF-1 RGG, both of which are extensively studied IDPs known to undergo phase separation.^52,53^ For these simulations, we employed the HPS-Urry model^54^ which has been previously documented for capturing the conformational properties and phase behavior of a wide range of IDPs. Recent research has further demonstrated its capability in capturing local conformational properties.^55^ By employing this model, which reproduced experimental *R*_g_ values, we observed the formation of a single droplet with several chains dispersed in the dilute phase (Figure S6). Notably, for these two natural proteins, chains exhibited a more collapsed conformation at the interface compared with the expanded structures within the droplet’s interior (Figure 5). These findings underscore the applicability of our conclusions derived from the homopolymer model to naturally occurring IDPs.

Recently, we discovered a strong correlation between the surface tension and the dilute phase (single-chain) *R*_g_ for charged disordered proteins.^13^ In future research, exploring the connection between conformations and dynamic properties at the interface will be interesting. Establishing such connections could significantly enhance our understanding of the biological function of biomolecular condensates.

**Figure 5:**
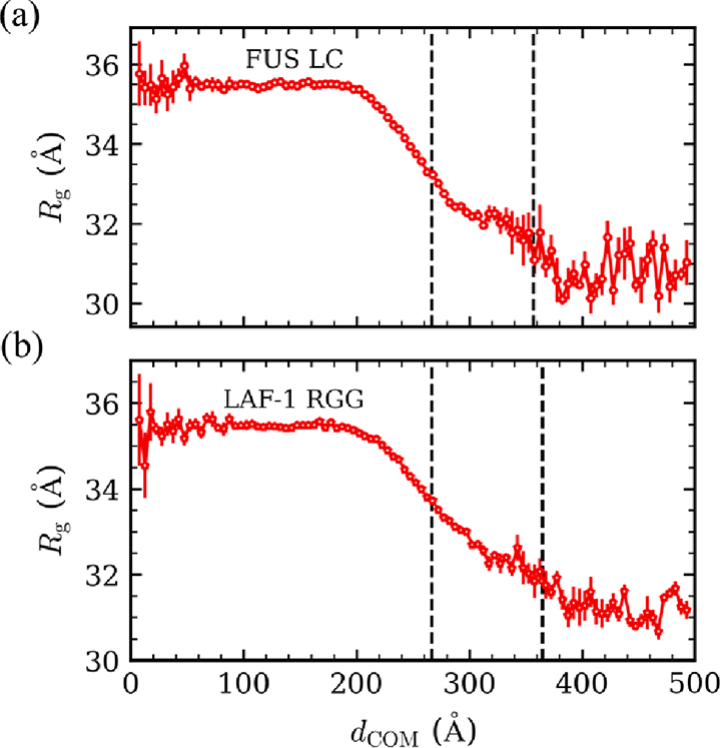
*R*_g_ as a function of *d*_COM_ for (a) FUS LC and (b) LAF-1 RGG sequences. The black dashed lines represent the boundaries of the interface.

## Supporting Information Available

The details of the model and simulation, method of average angle calculation, method of relative shape anisotropy calculation, sequences for natural proteins (FUS LC and LAF-1 RGG), probability distribution of *R*_g_, average *R*_g_ and *R*_e_, angle of different segments at different positions, and concentration distribution of natural proteins (PDF)
