# Supplementary material for: Conformational Properties of Polymers at Droplet Interfaces as Model Systems for Disordered Proteins
Source: ACS Macro Lett. 2023 Oct 19;12(11):1472–8. doi: 10.1021/acsmacrolett.3c00456 (PMC10771815; doi:10.1021/acsmacrolett.3c00456)
Supplement: Supplementary file 1 — mz3c00456_si_001.pdf [file mz3c00456_si_001.pdf]

Conformational Properties of Polymers at Droplet Interfaces as Model Systems for Disordered Proteins

Jiahui Wang,[1] Dinesh Sundaravadivelu Devarajan,[1] Arash Nikoubashman,[2,3,4*] and Jeetain Mittal[1,5,6,*]

[1]Artie McFerrin Department of Chemical Engineering, Texas A&M University, College Station, TX 77843, United States

[2]Institute of Physics, Johannes Gutenberg University Mainz, Staudingerweg 7, 55128 Mainz, Germany

[3]Leibniz-Institut für Polymerforschung Dresden e.V., Hohe Straße 6, Dresden 01069, Germany

[4]Institut für Theoretische Physik, Technische Universität Dresden, Dresden 01069, Germany

[5]Department of Chemistry, Texas A&M University, College Station, TX 77843, United States

[6]Interdisciplinary Graduate Program in Genetics and Genomics, Texas A&M University, College Station, TX 77843, United States

[*]Corresponding author email: anikouba@ipfdd.de, jeetain@tamu.edu

Simulation Details

We have used a coarse-grained model, in which each monomer is represented by a spherical bead that is connected to neighboring beads via a harmonic spring:

$$U_{\mathrm{b}}(r) = \frac{k_{\mathrm{b}}}{2}(r - r_0)^2, \tag{1}$$

where r is the distance between two bonded beads, k_{b}= 20 $\mathrm{kcal}/(\mathrm{mol}\text{Å}^2)$ is the spring constant and r_0 = 3.8 Å is the equilibrium length. The van der Waals interactions between nonbonded beads were modeling using a modified Lennard-Jones (LJ) potential[1]

$$U_{\mathrm{vdW}}(r) = \begin{cases} U_{\mathrm{LJ}}(r) + (1-\lambda)\varepsilon, & r \leq 2^{1/6}\sigma \\ \lambda U_{\mathrm{LJ}}(r), & otherwise \end{cases}, \tag{2}$$

where U_{LJ} is the standard LJ potential

$$U_{\mathrm{LJ}}(r) = 4\varepsilon\left[\left(\frac{\sigma}{r}\right)^{12} - \left(\frac{\sigma}{r}\right)^{6}\right], \tag{3}$$

with average hydropathy $\lambda = 0.865$ and bead diameter $\sigma = 6.46$ Å for the homopolymer systems. The interaction strength was fixed to ε = 0.2 kcal/mol in all simulations which, in conjunction with other model parameters, was previously found to reproduce the experimentally measured R_{g} values of several IDPs. For computational efficiency, the pair potential U_{vdW} and its associated forces were truncated to zero at a distance of 4 σ.

To have good statistics, droplet simulations were performed by placing 5000 chains (50 beads per chain) into a cubic box of edge length 1010 Å with periodic boundary conditions in all directions. We performed Langevin dynamics simulations at temperature $T = 300$K. The friction

coefficient was set as $\gamma_i = m_i/t_{\mathrm{damp}}$, where m_i = 163.2 g/mol and t_{damp} = 1000 fs. Simulations were performed for 2.5 μs with a time step of 10 fs using HOOMD-blue[2] (ver. 2.9.3) with features extended using azplugins[3] (ver. 0.10.2). The simulation trajectories were saved every 1 ns. Analysis was based on the last 1.5 μs.

Bulk system simulations were performed by placing 500 chains into a cubic box at a constant pressure of P = 0 atm for 0.5 μs. After the chains achieved their preferred bulk-phase concentration, the simulation was performed using Langevin dynamics at constant volume for 1 μs. All the simulations were implemented at a fixed temperature of T = 300 K. The analysis was based on the last 0.5 μs. Other simulation settings were the same with droplet simulation.

Single chain simulations were performed by placing one chain into a cubic box of edge length 160 Å. Langevin dynamics simulations were performed for 1 μs at constant temperature $T =$ 300 K. The analysis was based on the last 0.5 μs. Other simulation settings were the same with droplet simulation.

Natural protein simulations were performed by placing 1500 chains into a cubic box of edge length 1000 Å with periodic boundary conditions in all directions. Langevin dynamics simulations were performed at temperature $T = 300$K for FUS LC and $T = 260$K for LAF-1 RGG. The hydropathy values (derived based on experimental transition temperatures) for the residues in natural proteins were set according to the HPS-Urry model[4]. The friction coefficient was set as $\gamma_i = m_i/t_{\mathrm{damp}}$, where m_i was the residue mass and t_{damp} = 1000 fs. Simulations were performed for 1 μs and other simulation settings were the same with droplet forming simulation.

Natural protein sequences used in this work

FUS LC (length = 163)

MASNDYTQQATQSYGAYPTQPGQGYSQQSSQPYGQQSYSGYSQSTDTSGYGQSSYSSY
GQSQNTGYGTQSTPQGYGSTGGYGSSQSSQSSYGQQSSYPGYGQQPAPSSTSGSYGSSS
QSSSYGQPQSGSYSQQPSYGGQQQSYGQQQSYNPPQGYGQQNQYNS

LAF-1 RGG (length = 168)

MESNQSNNGGSGNAALNRGGRYVPPHLRGGDGGAAAAASAGGDDRRGGAGGGGYRR
GGGNSGGGGGGGYDRGYNDNRDDRDNRGGSGGYGRDRNYEDRGYNGGGGGGGNRG
YNNNRGGGGGGYNRQDRGDGGSSNFSRGGYNNRDEGSDNRGSGRSYNNDRRDNGGD
G

Average angle calculation for isotropic distribution of segments

The average angle of segments distributed without any preferred orientation can be calculated as

$$\theta_{\mathrm{ave}} = \int_{\pi/2}^{\pi} \theta sin\theta \, d\theta = \pi - 1, \qquad (4)$$

where θ is the angle between the segment-to-droplet COM vector and the segment end-to-end vector.

Relative shape anisotropy (κ^2) calculation for droplet:

By considering droplet as one long polymeric chain, κ^2 can be calculated using eigenvalues (λ) of the gyration tensor:

$$\mathrm{G} = \frac{1}{N}\sum_{i=1}^{N} \Delta \mathrm{r_i} \Delta \mathrm{r_i}, \qquad (5)$$

$$\kappa^2 = 1 - 3\,\frac{(\lambda_1\lambda_2+\lambda_2\lambda_3+\lambda_3\lambda_1)}{(\lambda_1+\lambda_2+\lambda_3)^2}, \qquad (6)$$

where $\mathbf{r}_i$ is the vector from the chain's mass center to residue i, and N is the total number of monomers in the droplet.

Supplementary Figures

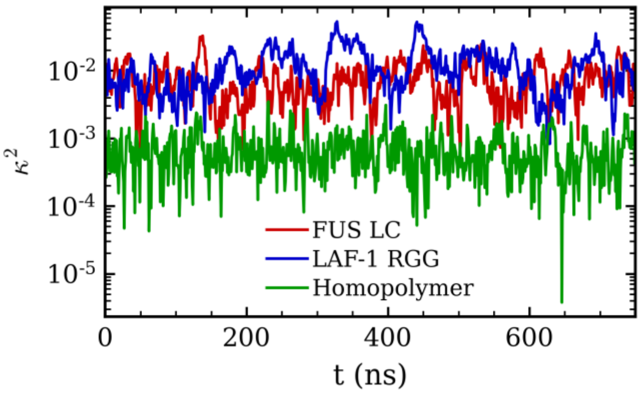

Fig. S1. Relative shape anisotropy (κ^2) of the droplets with respect to time for homopolymer, FUS LC, and LAF-1 RGG.

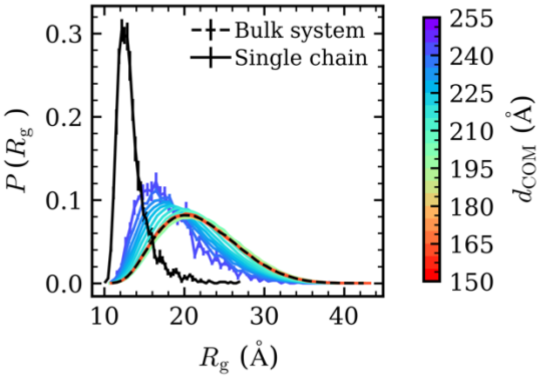

Fig. S2. Distribution of radius of gyration (R_g). The black dashed line represents the $R_g$distribution in the bulk system. The black solid line represents the R_g distribution of a single chain. The color gradient from red to purple corresponds to the distribution of R_g for chains with d_{COM} ranging from 150 to 255 Å.

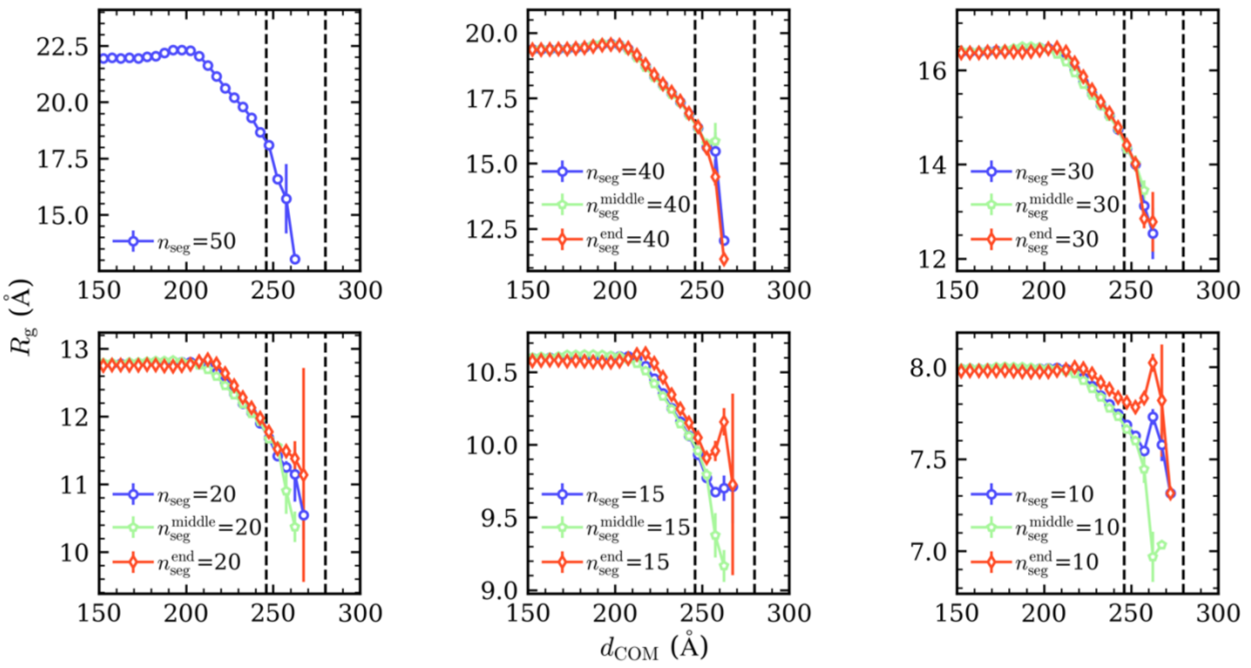

Fig. S3. R_g of chains and segments consisting of 40, 30, 20, 15, and 10 monomers as a function of d_{COM}. The purple line represents the average R_g for all segments. The green line represents segments located in the middle of the chain, while the red line represents segments located at the chain's ends. The black dashed lines represent the boundaries of the droplet interface.

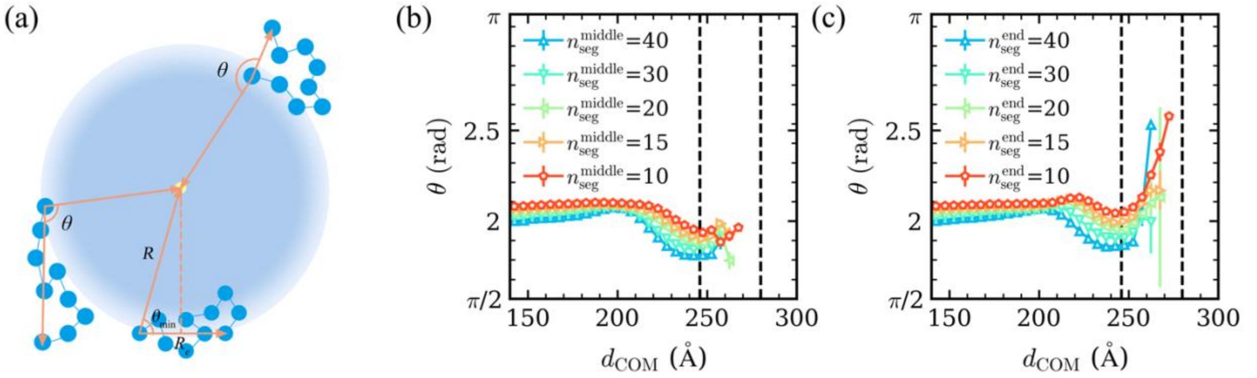

Fig. S4. Orientation calculation. (a) Schematic of calculation method for angle (θ) and the minimum of θ (θ_{min}). (b) θ of the segments of different length located at the middle of a chain as a function of d_{COM}. (c) θ of the segments of different length located at the ends of a chain as function of d_{COM}.

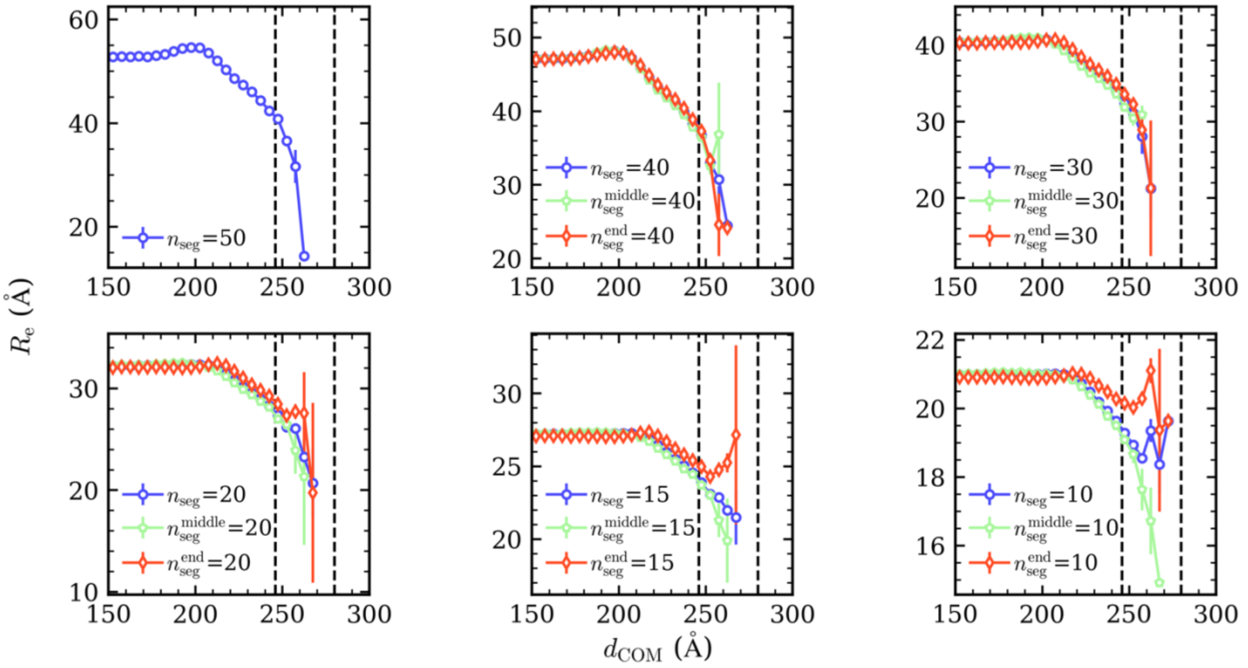

Fig. S5. End-to-end distance (R_e) of chains and segments consisting of 40, 30, 20, 15, and 10 monomers as a function of d_{COM}. The purple line represents the average R_e for all segments. The green line represents segments located in the middle of the chains, and the red line represents segments located at the chain's ends.

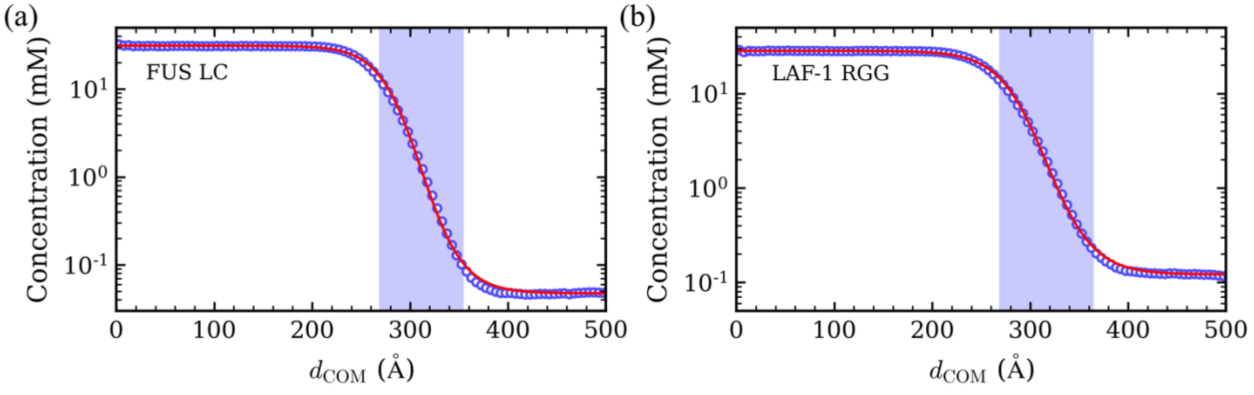

Fig. S6. Concentration as a function of distance from the droplet's center of mass (d_{COM}) for (a) FUS LC and (b) LAF-1 RGG. The red line is the fitted curve for the simulation data (symbols). The purple shaded area is the interface region.

REFERENCES

(1) Ashbaugh, H. S.; Hatch, H. W. Natively Unfolded Protein Stability as a Coil-to-Globule Transition in Charge/Hydropathy Space. *J. Am. Chem. Soc.* **2008**, *130* (29), 9536–9542. https://doi.org/10.1021/ja802124e.
(2) Anderson, J. A.; Glaser, J.; Glotzer, S. C. HOOMD-Blue: A Python Package for High-Performance Molecular Dynamics and Hard Particle Monte Carlo Simulations. *Comput. Mater. Sci.* **2020**, *173*, 109363. https://doi.org/10.1016/j.commatsci.2019.109363.
(3) Https://Github.Com/Mphowardlab/Azplugins.
(4) Regy, R. M.; Thompson, J.; Kim, Y. C.; Mittal, J. Improved Coarse-Grained Model for Studying Sequence Dependent Phase Separation of Disordered Proteins. *Protein Sci.* **2021**, *30* (7), 1371–1379. https://doi.org/10.1002/pro.4094.
